# Clinical results after arthroscopic reconstruction of the posterolateral corner of the knee: A prospective randomized trial comparing two different surgical techniques

**DOI:** 10.1007/s00402-022-04403-7

**Published:** 2022-03-27

**Authors:** Sebastian Weiss, Matthias Krause, Karl-Heinz Frosch

**Affiliations:** 1 Department of Trauma and Orthopaedic Surgery, University Medical Center Hamburg-Eppendorf, Martinistr. 52, 20246 Hamburg, Germany; 2 Department of Trauma Surgery, Orthopaedics and Sports Traumatology, BG Klinikum Hamburg, Hamburg, Germany

**Keywords:** Posterolateral corner, Arthroscopy, PLC, Reconstruction, Knee, Posterior cruciate ligament

## Abstract

**Introduction:**

Arthroscopic reconstruction techniques of the posterolateral corner (PLC) of the knee have been developed in recent years. Reconstruction techniques for higher-grade PLC injuries have not yet been validated in clinical studies. This study aimed to compare clinical outcomes of two different techniques and to present results of the first prospective randomized clinical trial of patients to undergo these novel procedures.

**Materials and methods:**

19 patients with Fanelli Type B posterolateral corner injuries and additional posterior cruciate ligament ruptures were included in this prospective study. They were randomly assigned to one of two novel arthroscopic reconstruction techniques, based on open surgeries developed by *Arciero* (group A) and *LaPrade* (group B). Follow-up was conducted at 6 and 12 months postoperatively and included clinical examinations for lateral, rotational and posterior stability, range of motion and subjective clinical outcome scores (IKDC Subjective Score, Lysholm Score, Tegner Activity Scale and Numeric Rating Scale for pain).

**Results:**

At 6 and 12 months postoperative, all patients in both groups presented stable to varus, external rotational and posterior forces, there were no significant differences between the two groups. At 12-month follow-up, group A patients showed significantly higher maximum flexion angles (134.17° ± 3.76° vs. 126.60° ± 4.22°; *p* = 0.021) compared to patients of group B. Duration of surgery was significantly longer in Group B patients than in group A (121.88 ± 11.63 vs. 165.00 ± 35.65 min; *p* = 0.003). Posterior drawer (side-to-side difference) remained more reduced in group A (2.50 ± 0.69 mm vs. 3.27 ± 0.92 mm; *p* = 0.184). Subjective patient outcome scores showed no significant differences between groups (Lysholm Score 83.33 ± 7.79 vs. 86.40 ± 9.21; *p* = 0.621).

**Conclusions:**

This study indicates sufficient restoration of posterolateral rotational instability, varus instability and posterior drawer after arthroscopic posterolateral corner reconstruction without neurovascular complications.

Increased postoperative range of motion and a shorter and less invasive surgical procedure could favor the arthroscopic reconstruction technique according to Arciero over LaPrade’s technique in future treatment considerations.

## Introduction

The posterolateral corner of the knee consists of the lateral/fibular collateral ligament (LCL/FCL) and the popliteus complex (PTC). The popliteus complex itself contains the popliteus muscle tendon unit (PLT) and the arcuate complex (AC), which is formed by the popliteofibular ligament (PFL), the fabellofibular ligament and the popliteomeniscal fibers [1]. It has a highly complex stabilizing function against various forces to the knee. The popliteus complex serves as the most important static and dynamic stabilizer against external tibial rotation and posterior tibial translation [2–4] while the lateral collateral ligament provides stability against varus forces [5].

In recent years, arthroscopic reconstruction techniques have been developed to provide stability while utilizing the benefits of arthroscopic compared to open surgeries [6]. Advantages of arthroscopic procedures include a better visualization of anatomical landmarks for drill tunnel placements, minimal soft tissue damage, lower infection rates and especially a better protection of the common peroneal nerve since its preparation and visualization is obsolete [1].

For lower-grade instabilities (Fanelli Type A, PoLIS LI-A) [7], anatomic reconstruction of the popliteus complex (popliteus bypass) has shown promising results [8–10]. Novel arthroscopic techniques for anatomical reconstruction in higher-grade instabilities (Fanelli Type B or C, PoLIS LI-B or LI-C [7]), based on *Arciero’s* and *LaPrade’s* procedures, have recently been described by Frings et al., and Kolb et al. [11–13]. In a biomechanical evaluation of an arthroscopic reconstruction according to Arciero, a nearly normal stability of the knee could be restored [13].

So far, clinical results of these reconstruction techniques remained elusive. In this study, we aimed to detect differences in clinical outcomes of arthroscopic anatomical PLC reconstruction techniques described by Frings et al.. and Kolb et al.. and to present results of the first prospective randomized clinical trial of patients to undergo these procedures.

We hypothesized that both procedures can sufficiently restore posterior, lateral and external rotational stability in high-grade posterolateral corner injuries and that there would be no differences in clinical outcome between the two groups.

## Material and methods

This study was approved by the local ethics committee of the regional medical association (PV7212). Informed consent was obtained by each patient in this study.

### Preoperative assessment

Patients who presented to our clinic between years 2018–2020 with high-grade injuries (Fanelli Type B, PoLIS LI-B [7]) to the posterolateral corner and consent to participate were included in this study. Inclusion criteria were chronic injuries (> 3 weeks) with a combined varus and posterolateral rotational instability and additional posterior instability due to injury to the posterior cruciate ligament. Patients with higher-grade posterolateral corner injuries and additional affected structures (biceps femoris muscle tendon rupture, iliotibial band injury, fracture of the fibular head, etc.) were excluded from this study, as they required additional open surgery to address these specific structures. Fanelli type A injuries were excluded as well.

Preoperative clinical evaluation was performed by an experienced and fellowship trained orthopedic surgeon and included the posterior drawer test in neutral, internal, and external rotation in 30° and 90° of knee flexion (dial test); lateral and medial gapping under varus and valgus stress opening in full extension and 20°–30° of flexion. Stress radiographs in 90° of flexion and neutral rotation were performed on the injured and non-injured knee to objectify the side-to-side difference of the posterior translation. Additionally, all patients underwent magnetic resonance imaging of the knee.

### Surgical procedure

**Fig. 1:**
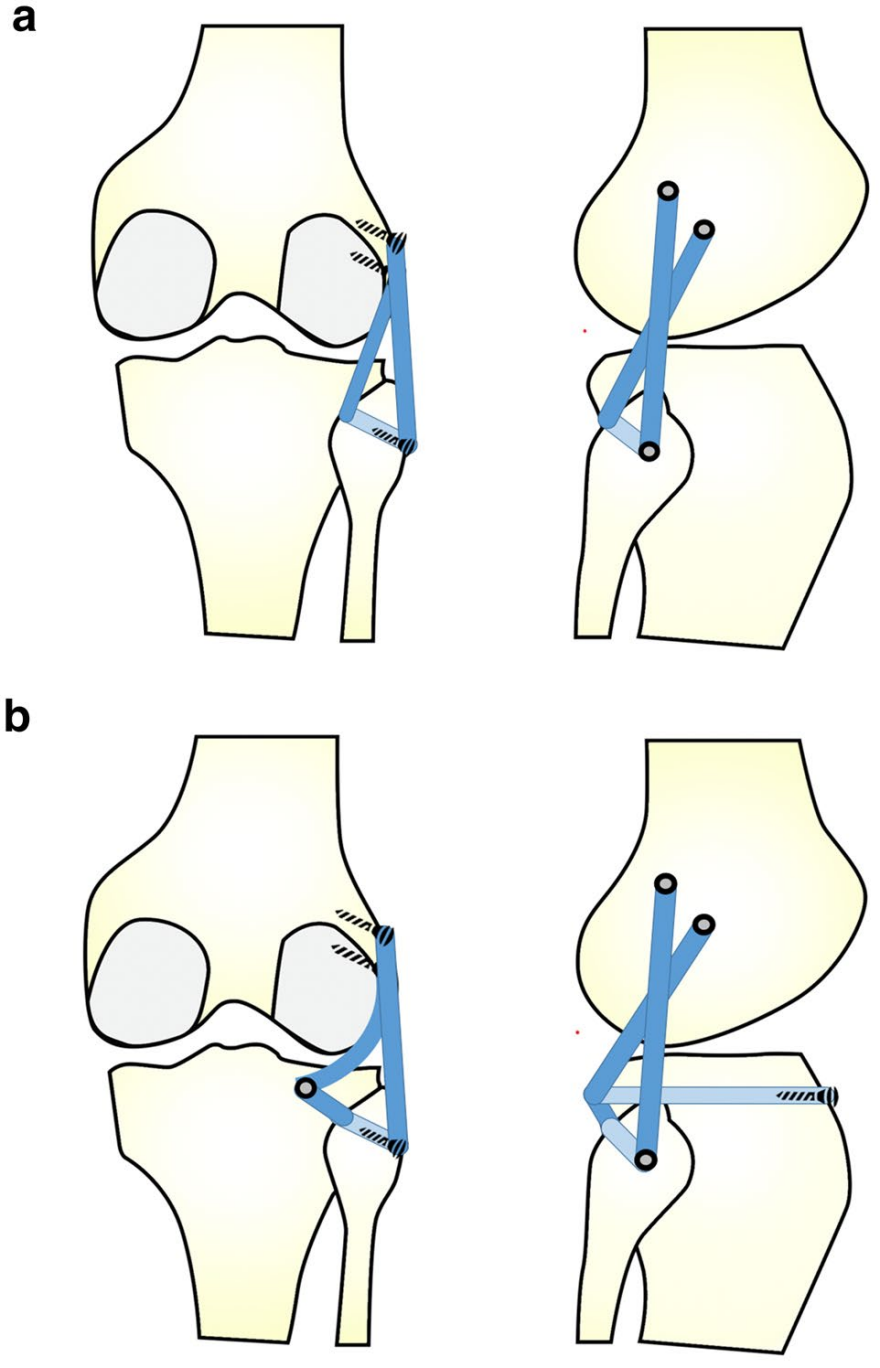
Schematic drawings of the two different techniques for posterolateral corner reconstruction in posterior (left) and lateral view (right). **a** Fibula-based, single-graft reconstruction technique based on *Arciero’s* technique for open PLC reconstruction (group A). **b** Tibio-fibular-based, double-graft technique according to *LaPrade’s* procedure (group B)

The type of surgical procedure was determined by block randomization, dividing patients into two subgroups. Surgeries were conducted strictly according to arthroscopic posterolateral corner reconstruction techniques described by Frings et al. [11] (Arciero’s technique, group A) and Kolb et al. [12] (LaPrade’s technique, Group B) (Fig. 1). Surgeries were all performed by the same surgeon with high expertise in arthroscopic knee surgery. Additional PCL reconstruction was performed arthroscopically during the same procedure [14]. The duration of the surgery was evaluated (starting from skin incision, including tendon harvest and all arthroscopic procedures until skin closure). Furthermore, length of stay as a hospital inpatient and surgery-related complications were investigated.

### Rehabilitation

The first 2 weeks after surgery patients wore a PCL brace (Jack PCL Brace; Albrecht, Bernau am Chiemsee, Germany) with a maximum flexion of the knee of 20°. Passive flexion of the knee was performed up to 40° of flexion in prone position by physical therapists after drain removal. Patients were allowed partial weight bearing of 20 kg on crutches for 6 weeks.

In weeks 3 and 4, patients were passively mobilized up to 60° of flexion and then to 90° in weeks 5 and 6 while increasing weight bearing to half of their body weight. The brace was recommended with limited range-of-motion for 6 weeks (day and night) and for another 6 weeks without limited range-of-motion.

After 6 weeks, patients were allowed full range of motion and started full weight bearing. Additionally, active flexion of the knee against gravity and cycling was allowed.

Forced flexion against resistance was allowed after 12 weeks, simultaneously jogging was permitted. Stop-and-go sports were allowed depending on individual return-to-play and return-to-competition tests after 6–9 months.

### Follow-up

Follow-up was conducted at 6 and 12 months after surgery by a single author, who was blinded to the type of surgical procedure to avoid bias. Clinical examination of range of motion and external rotation in 30° and 90° of flexion was performed with the use of a HALO Goniometer® (HALO, model HG1, HALO Medical Devices, Australia) [15]. Posterior drawer tests were measured with a Rolimeter® in the technique according to Höher et al. [16]. Varus instabilities were examined in 30° of knee flexion. All examinations were also performed on the contralateral knee and parameters were evaluated as side-to-side differences.

Subjective scores included the IKDC subjective score, the Lysholm Score and Tegner Activity Scale (TAS) as well as a Numeric Rating Scale (NRS) for current pain at time of follow-up. All of the above subjective scores are validated and were presented in the German version, as it was the native language of all included patients [17–19].

### Statistical analysis

Statistical analysis was performed using SPSS 26.0 (SPSS Inc, Chicago, IL). Mean values ± standard deviations were reported for all parameters. Statistical tests were performed as Mann–Whitney test, all tests were non-parametric test and statistical significance was set at *p* < 0.05. As this study was designated as a pilot study of rare injuries, power analysis was not applicable.

## Results

19 patients were included in this study, four patients were lost to follow-up for geographical reasons.

All patients in both groups initially presented with chronic Fanelli Type B injuries with varus opening up to 10 mm, an increased external rotational instability in dial test compared to the contralateral knee and subjective instability. There were not relevant comorbidities in patients of either group which would have influenced patient outcomes, as higher-grade instabilities were not included and there was no preexistence of arthrofibrosis. There was no significant difference in patient age at the time of surgery (*p* = 0.356) or the extent of preoperative posterior drawer as side-to-side difference between groups A and B. Duration of surgery was significantly longer in Group B patients than in group A (165.00 ± 35.65 vs. 121.88 ± 11.63 min; *p* = 0.003). There was no significant difference in the duration of in-hospital stay and one surgery-related complication was present in each group (Table 1). There were no neurovascular complications of the surgical procedures.

**Table 1:** Patient demographics and treatment parameters

	Group A	Group B	*p*-Value^1^
Age (years)	40.22 ± 11.49	35.38 ± 11.01	0.3559
Sex (male/female)	7/4	7/1	–
Duration of surgery (min)	121.88 ± 11.63	165.00 ± 35.65	**0.003**
Length of stay in hospital (days)	5.34 ± 1.51	5.25 ± 0.89	0.753
Complications	Dislocation of femoral PCL graft button (*n* = 1)	Arthrofibrosis requiring revision surgery (*n* = 1)	–

All values are listed as mean value ± standard deviation

^1^Values < 0.05 were considered statistically significant and are highlighted in bold letters

All patients in both groups received additional PCL reconstruction during the same surgical procedure.

At 6-month follow-up, patients of group A showed similar flexion angles as group B, a slightly reduced remaining posterior drawer, a larger external rotation deficit at 30° of knee flexion and a smaller external rotation deficit at 90° of knee flexion compared to group B, all measured as side-to-side differences.

**Fig. 2:**
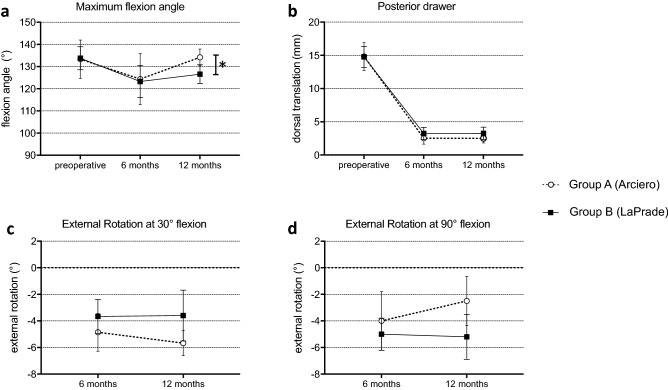
Clinical results at 6- and 12-month follow-up. **a** Maximum flexion angles of the injured knees. Flexion angles at 12 months postoperative were significantly higher in group A (indicated by asterisk, *p* = 0.021). **b** Posterior drawer measured via Rolimeter ® as side-to-side difference to the contralateral knee. **c** External rotation at 30° of knee flexion, measured as a side-to-side-difference. **d** External rotation at 90° of knee flexion, measured as a side-to-side-difference. No other parameter showed statistically significant differences between the two groups at any given time point. Graphs are drawn as mean values ± standard deviation

At 12 month follow-up, group A patients showed significantly better maximum flexion angles (134.17° ± 3.76° vs. 126.60° ± 4.22°; *p* = 0.021) compared to patients of group B. Posterior drawer (side-to-side difference) remained more reduced in group A compared to the preoperative state (2.50 ± 0.69 mm vs. 3.27 ± 0.92; *p* = 0.184). Group B showed less restraints in external rotation at 30° of flexion and slightly more restraints at 90° of flexion (Fig. 2). The patient of group B who showed arthrofibrosis had a preoperative range-of-motion of 130° (extension/flexion: 0–0–130°) and postoperative range-of-motion of 105° (extension/flexion: 0–5–110°) and was the only patient to present with an extension deficit.

All patients of both groups presented stable under varus stress at 6- and 12-month follow-up.

Aside from maximum flexion angles at 12 months, none of the above listed differences between the two groups showed statistical significance (Table 2).

**Table 2:** Cl**i**nical examination results at follow-up

	Group A	Group B	*p*-Value^1^
Flexion angle preoperative (°)	133.30 ± 8.66	133.75 ± 5.18	0.870
Flexion angle 6 months (°)	124.38 ± 11.48	123.29 ± 7.23	0.464
Flexion angle 12 months (°)	134.17 ± 3.76	126.60 ± 4.22	**0.021**
Posterior drawer preoperative^a^ (mm)	14.82 ± 2.09	14.75 ± 1.58	0.959
Posterior drawer at 6 months^a^ (mm)	2.52 ± 0.88	3.24 ± 0.93	0.248
Posterior drawer at 12 months^a^ (mm)	2.50 ± 0.69	3.27 ± 0.92	0.184
External rotation at 30° of flexion at 6 months^a^ (°)	− 4.86 ± 3.67	− 3.67 ± 3.14	0.798
External rotation at 30° of flexion at 12 months^a^ (°)	− 5.67 ± 2.34	− 3.60 ± 4.28	0.509
External rotation at 90° of flexion at 6 months^a^ (°)	− 3.92 ± 5.83	− 5.00 ± 2.97	0.815
External rotation at 90° of flexion at 12 months^a^ (°)	− 2.40 ± 4.51	− 5.20 ± 3.77	0.260

None of the evaluated parameters showed statistical significance (*p* < 0.05)

^a^Measured as side-to-side difference

^1^Values < 0.05 were considered statistically significant and are highlighted in bold letters

**Fig. 3:**
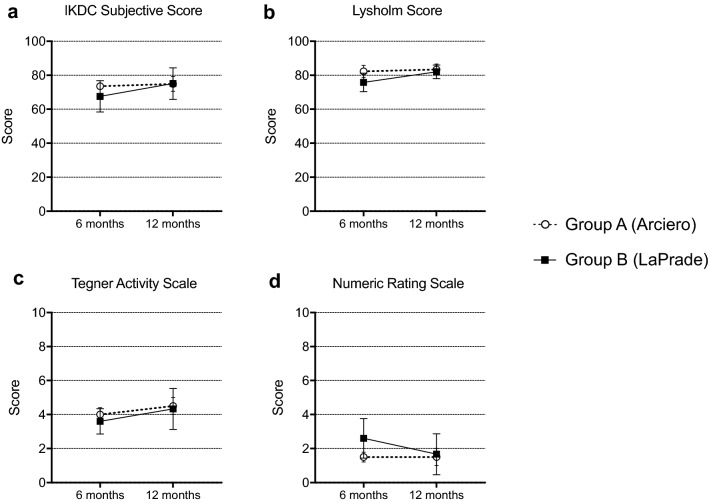
Clinical subjective scores at 6- and 12-month follow-up. **a** IKDC subjective score (range 0–100). **b** Lysholm score (range 0–100). **c** Tegner Activity Scale (range 0–10). **d** Numeric Rating Scale (range 0–10), describing current average pain in the injured knee at time of follow-up. No parameter showed statistically significant differences between the two groups at any given time point. Graphs are drawn as mean values ± standard deviation

Subjective scores showed a higher IKDC Subjective Score and Lysholm Score, as well as lower average pain in the injured knee at 6-month follow-up in Group A. None of these differences proved to be statistically significant (Fig. 3).

All other subjective parameters only showed minor differences between groups (Table 3). Only one patient in each group reported intermittent subjective instability, all other patients described their knee as subjectively stable.

**Table 3:** Patient Outcome scores at follow-up

	Group A	Group B	*p*-Value^1^
IKDC score at 6 months	74.49 ± 3.18	67.56 ± 20.63	> 0.999
IKDC score at 12 months	74.92 ± 10.78	79.56 ± 14.13	0.567
Lysholm score at 6 months	82.20 ± 6.18	75.81 ± 12.15	0.381
Lysholm score at 12 months	83.33 ± 7.79	86.4 ± 9.21	0.621
Tegner Activity Scale at 6 months	4.0 ± 0.7	3.6 ± 1.7	> 0.999
Tegner Activity Scale at 12 months	4.5 ± 1.3	4.4 ± 1.5	0.961
Pain level (NRS) at 6 months	1.6 ± 0.6	2.6 ± 2.6	0.643
Pain level (NRS) at 12 months	1.5 ± 1.2	1.2 ± 1.6	0.706

None of the evaluated parameters showed statistical significance (*p* < 0.05)

^1^Values < 0.05 were considered statistically significant

## Discussion

The most important findings of the present study were that all patients in both groups showed sufficient restoration of posterior, rotational and lateral joint stability in clinical examinations at both follow-up time points, as it was hypothesized in the aim of this study. Only two patients reported intermittent subjective instability which was not confirmed in objective clinical examinations and may be contributed to remaining muscular deficits. As restoration of objective and subjective joint stability was the primary indication for surgical treatment, arthroscopic reconstruction of the posterolateral corner in both techniques can be interpreted as successful.

In chronic injuries with high grade posterolateral rotational instabilities (Fanelli Type B or C, PoLIS LI-B or LI-C [7]), reconstruction has shown superior results compared to repair of injured structures [20–23]. While there are a variety of different surgical approaches to non-anatomic and anatomic reconstructions (fibular-/tibia-based, single-/multi-graft), anatomic reconstructions developed by Arciero et al. [24] and LaPrade et al. [25] have shown the most promising biomechanical and clinical results [26–28]. They are well established as open surgical procedures [29] and have shown equal restoration of joint stability in biomechanical studies [30].

As this study aimed to detect potential differences between the two surgical procedures, the only statistically significant outcome measure was the group A patients’ increased maximum flexion angle at 12 months postoperative as a relevant factor in judgement of functional outcomes. Aside from range of motion, only minor differences without statistical significance in objective and subjective outcome measures were found. Yet, further non-significant tendencies seemed to also show slightly better functional results in group A, displayed in postoperative posterior drawer and higher subjective outcome scores (IKDC, Lysholm, Tegner Activity Scale, NRS) after 6 months. These findings are in line with biomechanical studies of Treme et al., and Vezeridis et al. [30, 31], who showed similar results in restoration of joint stability against varus and external rotation forces with both surgical procedures. Drenck et al. [10] showed that reconstruction of the posterolateral corner via popliteus bypass graft, as it is performed in LaPrade’s procedure (group B), provides sufficient stability against external rotational forces. Clinical results of arthroscopic reconstruction in low-grade (Fanelli Type A) PLC injuries showed similar outcomes to open procedures [9].

This is also confirmed by our study as patients (in both groups) showed sufficient restoration of rotational stability with even a slight overconstraint in external rotation compared to the contralateral knee, yet there were no significant differences between the two groups.

Additionally to their clinical outcomes, both procedures need to be evaluated with regard to their invasiveness and risk of complications.

The surgical procedure took significantly longer in group B compared to group A patients and therefore is accompanied by an increased risk for general surgical complications (e.g., postoperative infection, pain, deep vein thrombosis) [32, 33]. It has to be considered that all patients received additional PCL reconstruction during the same surgery. As the PCL reconstruction technique was identical in every surgery, longer duration of surgery has to be attributed to the inherent differences of the two procedures.

*Arciero’s* technique [11] (group A) is a fibular-based, single-graft technique, while *LaPrade’s* technique [12] (group B) is a tibio-fibular-based, double-graft technique. The tibial fixation aspect in LaPrade’s technique aims to provide a more anatomic reconstruction but has also to be seen as a more invasive procedure. Additional preparation and establishment of the tibial drill tunnel presents additional trauma (and risk of collision with a tibial drill tunnel of the PCL graft), while the larger number of grafts increases scar tissue formation and therefore risk of reduced flexibility and arthrofibrosis [34, 35]. This aspect is confirmed by the reduced maximum flexion angles of group B patients after 12 months (126.60° ± 4.22° vs. 134.17° ± 3.76°; *p* = 0.021). One patient of group B in our study presented with arthrofibrosis that required revision arthrolysis surgery. The number of needed grafts is also highly relevant if patient autografts are used, as risk of donor site morbidity is increased with additional tendon harvests [36, 37]. While some experts in the field of PLC reconstruction, especially in North America, favorably use allografts, autografts are still the most widely used type of tendon graft [29].

With the above-mentioned differences between the two procedures, the procedure described by Frings et al. (*Arciero’s* procedure) appears to be less invasive while providing similar clinical results. The procedure described by Kolb et al. (*LaPrade’s* procedure) has the benefit of providing additional stability in cases of tibiofibular joint injuries.

Arthroscopic reconstruction of the posterolateral corner requires profound knowledge of anatomic relations, but has the advantage of allowing visualization of all key structures including the posteromedial aspect of the fibular head to place a fibular drill tunnel under visual instead of palpatory control [38, 39]. Aside from general benefits of arthroscopic compared to open surgery such as lower infection rates, less scar tissue, faster rehabilitation and less pain, this allows for improved fibular tunnel placement. While some experts have raised concerns that arthroscopic PLC reconstructions bear a higher risk of tunnel misplacement and neurovascular injuries [40, 41], our clinical results showed no neurovascular or tunnel-related complications. Studies have shown that development of a transseptal portal and posterolateral arthroscopy can be performed safely at intraoperative knee flexion of 90° [38, 42–44]. The absence of postoperative common peroneal nerve damage symptoms in our study suggests that arthroscopic fibular drill tunnel placement does not cause neurovascular damage as commonly as recent studies have described [45]. While lack of intraoperative complications could be attributed to the small size of our study group, the common peroneal nerve injury rate of up to 57% in open fibular drill tunnel placement, as presented by Hohmann et al., in a cadaveric study, appears to be unlikely from our perspective and is not reflected in our clinical experience. Certainly, success of arthroscopic PLC reconstruction is strongly dependent on the surgeon’s experience in the field of arthroscopic reconstructive knee surgery.

## Limitations

This study has several limitations. First of all, the size of our study group is limited, due to the rare nature of these injuries especially in the pandemic situation of the last 18 months. Differences between the groups have a higher risk of being coincidental, as statistical evaluation showed no significant differences in most outcome measures.

Furthermore, while inclusion and exclusion criteria were very precise, time between injury and surgery showed variability, but was always > 3 weeks and therefore considered as a chronic injury.

Follow-up clinical examinations were performed via Rolimeter® instead of stress radiographs. While evaluation of posterior drawer and lateral instability in the injured state is commonly performed with stress radiographs, follow-up examinations have shown equally reliable results with these devices [16].

## Conclusion

This study indicates sufficient restoration of posterolateral rotational instability, varus instability and posterior drawer after arthroscopic posterolateral corner reconstruction without neurovascular complications with an excellent radiographic and clinical outcome.

Increased postoperative range of motion and a shorter and less invasive surgical procedure could favor the arthroscopic reconstruction technique according to *Arciero* over *LaPrade’s* technique in future treatment considerations.
